# Genetic variation, brain, and intelligence differences

**DOI:** 10.1038/s41380-021-01027-y

**Published:** 2021-02-02

**Authors:** Ian J. Deary, Simon R. Cox, W. David Hill

**Affiliations:** Lothian Birth Cohorts group, Department of Psychology, University of Edinburgh, 7 George Square, Edinburgh, EH8 9JZ UK

**Keywords:** Psychology, Neuroscience, Genetics

## Abstract

Individual differences in human intelligence, as assessed using cognitive test scores, have a well-replicated, hierarchical phenotypic covariance structure. They are substantially stable across the life course, and are predictive of educational, social, and health outcomes. From this solid phenotypic foundation and importance for life, comes an interest in the environmental, social, and genetic aetiologies of intelligence, and in the foundations of intelligence differences in brain structure and functioning. Here, we summarise and critique the last 10 years or so of molecular genetic (DNA-based) research on intelligence, including the discovery of genetic loci associated with intelligence, DNA-based heritability, and intelligence’s genetic correlations with other traits. We summarise new brain imaging-intelligence findings, including whole-brain associations and grey and white matter associations. We summarise regional brain imaging associations with intelligence and interpret these with respect to theoretical accounts. We address research that combines genetics and brain imaging in studying intelligence differences. There are new, though modest, associations in all these areas, and mechanistic accounts are lacking. We attempt to identify growing points that might contribute toward a more integrated ‘systems biology’ account of some of the between-individual differences in intelligence.

## Individual differences in human intelligence

This article is about some new contributions toward understanding the aetiology of individual differences in human intelligence. The focus is on genetic variation and brain imaging-derived differences, including where those two sources overlap. For more than a century, the field of research that studies intelligence differences has had some controversies (Box 1). Notwithstanding these, research findings on intelligence have much consensus, based on robust findings. The first part of this article summarises some of the findings from which reductionist approaches—including brain imaging and genetics—to intelligence differences proceed.

Box 1 Some controversies and some consensuses in intelligence Here is an abbreviated litany of controversies about human intelligence differences. Galton [130], who suggested that cognitive capabilities might be general, normally distributed, and somewhat heritable (modern data show some support for these suggestions), was also, notoriously, an originator of eugenics, and invented that word (see www.ucl.ac.uk/provost/inquiry-history-eugenics-ucl). Spearman [4] discovered the positive matrix of correlations among cognitive performance assessments, and developed a two-factor theory of intelligence, which had ‘general intelligence’, which he called *g*, and specific abilities, which he called ‘*s*’. Researchers such as Thurstone [131] and Gardner [132] disagreed, and thought there were several separate intelligences. Thomson [133] produced an ingenious theory about how the positive matrix of cognitive test correlations might occur without there being a *g* factor. Whether *g* is found (it is a replicable statistical finding [5]) and what it means (which is not known) have been discussed since then. Henry Herbert Goddard imported Binet’s intelligence test (the first one to be invented) to the USA and is documented to have over- and mis-applied it (Zenderland) [134]. Gould [135] strongly criticised the *g* factor in intelligence—stating that it was a necessary outcome of the statistical analytic methods applied (which is incorrect)—and the association between intelligence and brain size in his famous book, *The Mismeasure of Man*. The book has been criticised for having got both of these wrong (Carroll) [136]. Flynn [137] found that intelligence test scores increased across the years and generations of the middle two quarters of the 20th century. However, Flynn also made it clear that this does not alter the within-cohort reliability, validity, and heritability of intelligence test scores. Nevertheless, the cause(s) of the ‘Flynn’ effect on intelligence test scores still remains mysterious. Herrnstein and Murray wrote a book called *The Bell Curve* [138]. They analysed data from the USA’s National Longitudinal Survey of Youth 1979 and found that higher intelligence in late adolescence/early adulthood was related to better life outcomes by the 30 s. The book was strongly and widely criticised, especially for its dealing with ethnic group differences, and for not having published its analyses via peer review.

Calming, consensual oil was poured on intelligence’s stormy waters in 1996, by the American Psychological Association (APA). As a result of the controversy caused by *The Bell Curve*, the APA put together a Task Force, chaired by cognitive psychology doyen Neisser [139], to tell non-experts what was (solidly) known and (as-yet) unknown about intelligence test score differences. The 11 persons in the Task Force—who became co-authors of an agreed article—surprised many. They were experts who were diverse in their viewpoints—for example, there were some individuals who were more environmentally inclined and some who were more genetically inclined, some who were associated with the hierarchical model of intelligence differences including *g* and some with different models of intelligence, etc. Yet, they wrote a still-useful article on some of the solid ground in intelligence research. Among many topics addressed, they recognised the prominence of the psychometric testing approach to intelligence differences, and the hierarchy with the *g* factor at the apex; they summarised the stability of intelligence test scores, their predictive validity for education, work and other life outcomes, their having environmental and genetic origins, the Flynn effect, and various types of group differences. The APA Task Force Report is still a must-read for obtaining a mostly disinterested and consensual summary about intelligence. Their list of intelligence’s unknowns are still mostly in that state; one of them was genetics, which we address here and which has moved on considerably. We recommend reading other, more recent summaries about knowns and unknowns in intelligence, though we wish to orient the reader that some come from more socially/environmentally inclined groups of authors (Nisbett et al.) [129], some from more genetically inclined (Gottfredson) [2], and two from one of the present authors (Deary) [27, 140]. Progress in finding social and environmental causes of intelligence has arguably been less successful than the biological research summarised herein, though the Nisbett et al. review discusses many growth points. Moreover, the Neisser et al., and Nisbett et al., summaries also deal with brain imaging and genetics, providing useful background to the present overview, as does Haier’s book, *The Neuroscience of Intelligence* [141]. Lest the reader makes the error of over-extending intelligence’s demesne and importance, the APA Task Force ended by emphasising—as do we—that there are many cognitive and non-cognitive aspects of human differences that are not captured by intelligence tests and general intelligence. *g* might be important, but it is far from being all that matters.

### Describing the phenotype of intelligence

We should make it clear to the reader that ‘intelligence’ is just one of the terms that are used to describe humans’ differences in thinking skills; others, sometimes used as near-synonyms, include cognitive ability, cognitive performance, cognitive functioning, and mental ability. Sometimes IQ (intelligence quotient) is used, although that has a specific meaning within the field of psychometrics. Intelligence (or the other terms listed in the previous sentence), as a human phenotype, is measured using cognitive tests, of which there are thousands. This hands the cynic a weapon that, to the ignorant, can glibly dismiss the field of research because, as Boring [1] famously wrote in 1923, “…intelligence as a measurable capacity must at the start be defined as the capacity to do well in an intelligence test. Intelligence is what the tests test.” That much-quoted last short sentence was not Boring’s opinion; rather, it was his saying that that is what one would think if one did not know the research findings. His next sentence starts, “This is a narrow definition, but it is the only point of departure for a rigorous discussion of the tests”. We shall have that rigorous discussion here. Before that, we offer another, much-cited definition: “Intelligence is a very general mental capability that, among other things, involves the ability to reason, plan, solve problems, think abstractly, comprehend complex ideas, learn quickly, and learn from experience. It is not merely book learning, a narrow academic skill, or test-taking smarts. Rather, it reflects a broader and deeper capability for comprehending our surroundings—‘catching on’, ‘making sense’ of things, or ‘figuring out’ what to do’ [2]. More succinctly, intelligence has been described as, “rapid and accurate problem solving” [3].

**Fig. 1:**
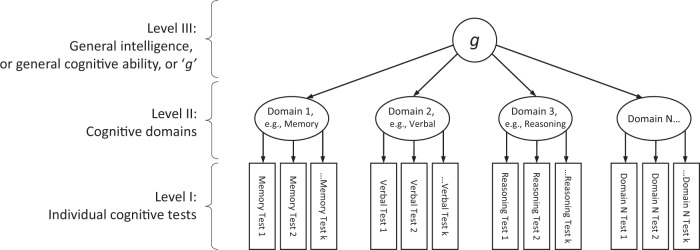
The hierarchical model of human intelligence differences. At the bottom, on level 1, individuals differ with regard to their performance on specific cognitive tests. Here we have shown that multiple tests (up to a number *k*) are used to assess each domain of cognitive capability. Scores on all tests of cognitive ability correlate positively. However, there are especially strong correlations among tests that tax the same cognitive domain (level 2). Level 2 illustrates three example cognitive domains: memory, processing speed, and verbal. There could be more (up to a number ‘*N*’), depending on the types of specific tests included in the battery. The names applied to domains are common-sense labels based on the apparently shared contents of the specific tests that contribute to them. It is possible to have tests that contribute to more than one domain (a possibility not shown in the Figure). Individual differences are observable at the domain level. However, people’s scores on any one cognitive domain correlate with their scores in other domains. This means that there is a third level describing the variance common across all the domains and, thereby, across all the individual cognitive tests. This is denoted, at Level 3, by general cognitive ability, general intelligence, or just ‘*g*’. It is important to note that this three-level structure emerges from the data and is not imposed on it. *g* tends to account for about 40% of the total test score variance when a battery of diverse cognitive tests is administered to a sample of people with a wide range of cognitive capabilities.

Cognitive ability differences form a hierarchy of variances. This grew from the finding that all cognitive tests are positively correlated; people who score well on one cognitive test tend to score well on all the others, no matter how different the cognitive skills being assessed appear to be. This finding has been replicated consistently since Charles Spearman discovered it in 1904 [4]. For example, John Carroll re-analysed correlation matrices of diverse cognitive tests from 400+ studies conducted in the 20th century [5]. These included studies by many of the most prominent researchers over that time, including those who had claimed not have found a general intelligence factor. Carroll found that, in all studies, the cognitive tests’ scores correlated positively, and that each study contained a general component that accounted for around 40%, sometimes more, of people’s differences in performance [5]. There was also variance at the level of cognitive domains, such as memory, reasoning, and speed; i.e., some cognitive tests correlate more highly with some tests—that have contents similar to theirs—than with others. And there was variance at the level of the individual tests. In summary, as shown in Fig. 1, the reasons that people do well on any cognitive test are that: they are generally intelligent; they are good at that type of test; that they are good at the specific skills in that test; and we should not forget the error in the measurement, and just having a good day for whatever reason.

General intelligence (*g*), as a statistical phenomenon, is a universal finding from different batteries of cognitive tests. However, there is still mystery about what causes the covariation. If a large sample of people take two different, diverse sets of cognitive tests, the two *g* factors from them correlate highly [6]; thus, *g* is not idiosyncratic, and its ranking of individuals does not differ substantially depending on the test battery. Among the sources of between-individual variance that are extractable from a set of cognitive test scores, it tends to be the *g* factor that leads the way in being associated with life outcomes, and with some possible origins, such as brain imaging indices and genetic variants. When researchers measure general intelligence they tend to use one of the following indices: ideally, the first unrotated principal component or factor from a battery of diverse cognitive tests; a total score from a test that has heterogeneous items covering diverse cognitive skills; or a test with a more homogeneous set of items that loads highly on the first unrotated principal component or general factor of several cognitive tests.

### Stability of intelligence differences and mean scores

Measures of general intelligence have high test-retest reliability. Taking an extreme example, the stability co-efficient of intelligence test scores is between 0.6 and 0.7 from age 11 years to about 80, even before correcting for measurement error [7].

The other type of stability—stability of age-related means—shows a well-reproduced pattern. Tests that involve the recall of learned information (called crystallised intelligence)—such as vocabulary, general knowledge, and some number skills—are relatively stable in mean levels from young adulthood to older age [8, 9]. Tests that involve active mental work (aspects of fluid intelligence) decline in mean levels from young or middle adulthood to older age [8, 9]. These include cognitive domains such as processing speed (e.g. in tests of coding numbers into symbols at speed), memory (e.g. delayed recall of a story or a list of words, or a working memory test, such as backward digit span), visuo-spatial ability (such as the replication of a 2-dimensional pattern using blocks in the Wechsler Block Design test), and abstract reasoning (such as the inductive reasoning from abstract patterns that is required in Raven’s Progressive Matrices). These more age-sensitive cognitive domains tend to age in concert, with half or more of the individual differences in their age-related declines accounted for by the ageing of general fluid intelligence [10]. There are individual differences in the ageing of intelligence, which has growing importance as populations have more older people who live longer [9]; that is, it is important to understand the determinants (especially the modifiable ones) of successful cognitive ageing.

### Predictive validity of intelligence: ‘healthy, wealthy, and wise’

Intelligence test scores at the end of primary schooling—at about 11 years of age—are highly correlated with educational outcomes several years later, whether that is measured as scores on standardised national examinations at 16 (where correlations up to 0.8 have been reported), years of education undertaken, or the highest qualification obtained [11, 12]. Probably, intelligence is causal to experiencing longer and more complex education, and there appears to be a small effect in the opposite direction too [13]. Thus, intelligence and education probably have a dynamic bi-directional, and possibly causal, association.

Intelligence is one of the best (and cheapest) predictors of performing well on a job, and of learning well on a job, with moderate correlations [14]. This applies to all levels of job complexity, though the correlations are somewhat higher with more complex occupations. Higher childhood intelligence is moderately related to moving upward in occupational status from one’s parents (usually father) [15]. Intelligence is one among many other variables that are associated with socioeconomic status differences in the UK [15, 16]. More affluent parental socioeconomic status and more education are among other variables that independently contribute; the former effect is relatively small, and it is not certain to what extent education acts as a proxy for prior intelligence.

There is a robust and consistently sized association between higher intelligence measured in childhood or youth and longer life and better health [17]. Studies on this topic include unusually impressive samples, including a country’s almost-whole year-of-birth population [17] and samples that contain up to millions of subjects [18, 19]. People with higher intelligence in early life are, up to several decades later, less likely to suffer from poor health and then die from all causes, and, specifically, from heart disease, stroke, respiratory disease, smoking-related cancers, digestive diseases, dementia, accidents, and suicide, among other causes. A typical effect size for this field—cognitive epidemiology—is that a standard deviation (15 IQ-type points) advantage in intelligence in youth is associated with 20–25% lower risk of these illness and mortality outcomes up to several decades later. Expressed as a correlation, the association between childhood cognitive test scores and all-cause mortality is typically between about 0.15 and 0.2.

Therefore, intelligence, as operationalised by cognitive test scores, has a robustly characterised phenotype, high test-retest stability, and some predictive validity for education, work, and health; all are contributions to broader construct validity. However, there is a lack when it comes to understanding why some people are more intelligent than others. We have written about these matters previously. A book-length treatment in 2000 [20] examined possible origins of intelligence differences in so called ‘elementary’ cognitive components, brain parameters, and genetic variation. There are robust associations between intelligence test scores and apparently simpler processing speed measures such as reaction times [21] and the psychophysical procedure called inspection time [22]. We do not focus on these here, because we judge that they afford less-tractable possible causes of some of the between-individual differences in intelligence than genetic variation and brain structure and functioning. We previously summarised genetics and brain imaging associations with intelligence test scores in 2010 [23]. However, at that time there were no genome-wide association studies (GWAS) of intelligence and, since then, brain imaging studies have larger samples, new brain parameters, and have been linked with molecular genetic studies.

## Intelligence differences and genetic variation

### Heritability and genetic architecture of intelligence differences

Twin and family studies report that genetic differences are associated with individual differences in intelligence test scores (Box 2). If studies from all ages are taken together, genetic differences account for about 50% (standard error [SE] about 2%) of the variation in intelligence [24]. Higher heritability (see Glossary) estimates are found in samples of adults (where it can be 70% or slightly more) than in children (where estimates as low as 20–30% have been reported) [24–27]. The finding that intelligence is heritable has been replicated across multiple data sets sourced from different countries and times [28]. Our emphasis herein is on results from the newer, DNA-based studies rather than on traditional twin and family studies.

DNA-based studies have shown that a pattern of hierarchical variance is evident at the genetic as well as the phenotypic level. Using genomic structural equation modelling [29] it was found that a genetic general factor explained, on average, 58.4% (SE = 4.8%, ranging from 9 to 95% for individual tests) of the genetic variance across seven cognitive tests in people with European ancestry. This provides some support for the idea that the phenotypic structure of intelligence is in part due to genetic effects that act on a general factor of intelligence and also at more specific cognitive levels.

Since 2011, the heritability of intelligence has been investigated by direct testing of DNA in large numbers of unrelated individuals [30]. This is mostly based on the testing of genetic variants called single nucleotide polymorphisms (SNPs) (see Glossary). The statistical-genetic method used to estimate heritability is called genome-based restricted maximum likelihood single component (GREML-SC) (Box 2). This tests how closely people’s similarity in cognitive test scores associates with their genetic similarity, the latter being based on hundreds of thousands of SNPs. In such studies, heritability estimates are about 20–30% (SEs < 1% in recent studies) [31, 32]. The lower estimates of heritability found using GREML-SC are probably due to the technique’s being better at capturing variance from genetic variants in linkage disequilibrium (LD) with common SNPs rather than those that are less common or in lower LD [33]. This difference in estimated heritability of intelligence between twin-based studies and DNA-based studies using SNPs has been recovered using DNA testing and the GREML-KIN analysis method in a large cohort of individuals that included families (Box 2) [32].

Non-additive genetic variation, including dominance and epistasis, has been postulated as a partial explanation for the gap in heritability estimates derived using twin and family methods compared with those derived using DNA-SNPs. However, one study found dominance effects were linked to less than 4% of phenotypic variance in complex traits [34]. Furthermore, quantitative genetics theory predicts that epistasis is unlikely to be associated with a substantial amount of phenotypic variance [35]. Moreover, the results of GREML-KIN have been replicated in unrelated individuals by deriving heritability estimates using high-quality imputation panels [32], indicating that non-additive genetic effects, if present for intelligence, are not a major contributing factor to intelligence differences.

Although GREML-KIN might recover some of the heritability that is attributable to genetic variants that are in poor LD with common genotyped SNPs, it cannot determine the proportion of the heritability estimate that is due to dynastic effects [36]. Dynastic effects include instances where the genotype of a parent is associated with the phenotype of the offspring, including via alleles that are not passed from parent to offspring; this has been termed genetic nurture [37]. Whereas the presence of dynastic effects does not indicate a bias in methods that also capture indirect genetic effects, it does however hamper efforts to understand how genetic differences can give rise to phenotypic differences. This is because the resulting heritability estimate is, potentially, a combination of direct genetic effects (genetic variation in an organism that is associated with phenotypic variation in the same organism) and indirect genetic effects (where genetic endowment of one organism is associated with the phenotype of another organism).

The presence of dynastic effects has been indicated for education. In one study, its SNP-based heritability estimate was 29.2% (SE = 4.4%) before indirect genetic effects were removed, and 17% (SE = 9.4%) afterwards [36]. Moreover, environmental influence on the heritability of education was suggested by finding that a polygenic score (see below) predicting education in non-adoptees accounted for twice the phenotypic variance of a polygenic risk score applied to adoptees [38]. The variance accounted for by a polygenic score captures both direct genetic effects and indirect, environmentally mediated, genetic effects. When predicting education with polygenic scores in adopted individuals, the link between the rearing environment provided by the genetically related parent and the phenotype of the offspring is broken. Because intelligence is highly genetically and phenotypically associated with education (see below), it appears likely that indirect genetic effects influence intelligence too.

Box 2 Heritability of intelligence: why different methods give different results Heritability describes the proportion (often expressed as a percentage) of phenotypic variation in a tested sample of people that can be accounted for by genetic variation [142]. Note that heritability estimates apply to a sample at a given time; the estimate might be different in other groups, and in the same group at other times. Often, in human studies, only additive genetic factors are considered. Different methods are used to estimate heritability of intelligence (and other phenotypes). They give different estimates. These are not contradictory; rather, they are a reflection of the sources of genetic variation to which the methods afford access, as we explain below.

**Twin and family methods**

Twin- and family-based estimates of the heritability use the expected proportion of alleles shared between the participants as the estimate of genetic variance within the sample. They can include comparisons between monozygotic twins and dizygotic twins, as well as studies that include families consisting of parents, siblings, and other relationships. In each instance, a genetic effect on a trait is inferred if individuals who are more genetically similar are also more similar in terms of their intelligence test score. For intelligence, substantial (50% or more, unless the study is of young children) heritability estimates are found in twin studies [24] and family studies [143]. We note that twin studies assume that dizygotic twins have just as similar shared environments as monozygotic twins, a potential limitation that does not affect DNA-based studies.

**Genome-based restricted maximum likelihood single component (GREML-SC)**

GREML-SC, sometimes referred to as ‘the GCTA method’, was the first DNA-based genetic method used to derive an estimate of heritability for intelligence [30]. As with twin and family-based methods, genetic similarity is compared with phenotypic similarity. However, genetic similarity is measured, rather than inferred, using a genomic relationship matrix constructed from genotyped common SNPs. Importantly, closely related individuals are excluded from the analysis (typically those who are more than 0.025 similar, i.e. closer than a second cousin). This is an attempt to ensure that the similarity in environment between family members is not captured by genetic the genomic relationship matrix, which can result in an inflation of the heritability estimate. Heritability estimates of intelligence that use GREML-SC have typically been between 20 and 30% [30, 31, 143]. One of the major assumptions of GREML-SC is that genetic similarity is uncorrelated with environmental similarity. Whereas this assumption has been found not to hold in some situations, the inflation of the resulting heritability estimate is thought to be negligible [144]. Furthermore, GREML-SC assumes an infinitesimal, or polygenic, model whereby the trait examined is associated with a very large number of variants each making an infinitesimal contribution to phenotypic variance. GREML-SC assumes that SNP effects are normally distributed as well as independent of LD (see Glossary), and inversely proportionate to minor allele frequency [33].

**Genome-based restricted maximum likelihood kinship (GREML-KIN)**

GREML-KIN was introduced to capture the effects of rarer and less common genetic variants that are not captured using GREML-SC. Importantly, whereas it uses the same genetic data as GREML-SC, GREML-KIN uses samples with a dense and known pedigree to derive additional matrices to capture additional sources of variance from genetic variants that are in poor LD with common genotyped SNPs and to control for the effect of environmental influences. When GREML-KIN was applied to the study of intelligence, 54% of intelligence test variation was accounted for [32]; therefore, the DNA-based heritability of intelligence was about the same as those derived using twins. GREML-KIN has the same assumptions as GREML-SC. In addition, GREML-KIN uses closely related individuals, and the data it is applied to must contain a sufficiently dense pedigree in order to prevent an inflation of the heritability estimates due to shared environmental influences between those closely related individuals.

### Genome-wide association studies (GWAS) of intelligence: finding loci

Heritability analyses suggest the presence of genetic influence on a trait. They do not indicate which genetic variants are associated with trait variation. Initially, candidate gene designs (see Glossary) were used to test for associations between genetic variants and intelligence test scores. However, these designs were underpowered and produced no replicable results [39]. The qualified exception is that possession of the e4 allele of the gene for Apolipoprotein E (*APOE*) is reliably associated with slightly lower cognitive function at older ages, accounting for around 1% of the variance [40, 41]. No other SNP-based genetic variant comes close to this effect size in accounting for intelligence differences. The association might occur because APOE is involved in neuronal repair, and there is more repairing to do—and probably more individual differences (variance) in neurodegeneration—at older ages. The field changed to the conducting of GWAS (see Glossary) that are agnostic regarding which, if any, loci are associated with the trait of interest. This was driven by the availability of affordable arrays of hundreds of thousands of SNPs covering the genome, alongside the collection of large samples sizes and the formation of multi-sample consortia.

The first GWAS of intelligence with *N* > 3000, from 2011, detected no significantly associated loci [30]. However, it included the first DNA-based (GREML-SC-derived) heritability estimate of intelligence and showed that genotyped SNPs do account—collectively—for some of its variation [30]. For the next 6 years, GWASs conducted on intelligence test scores were largely unsuccessful in identifying associated genetic loci [42–45]. In 2018, three studies, using substantially overlapping samples, attained sample sizes of over 200,000 participants and found hundreds of genetic loci significantly associated with intelligence [31, 46, 47].

The first of the three studies had a sample size of 248,428; it found 187 (172 novel when it appeared online) independent regions of the genome that were associated with intelligence [46]. A major contribution to this study was from the large UK Biobank sample’s short Verbal and Numerical Reasoning test (VNR; called the ‘fluid’ test by UK Biobank, which is a misnomer). This study used a meta-analytic method (MTAG) to combine data sets using different indices of cognitive ability, and including educational attainment to increase statistical power. However, whereas a proxy-phenotype approach was used previously to identify SNPs that showed a joint association with education and intelligence [48], the meta-analytic method used in MTAG is different because it was designed to detect genetic associations with the target trait of intelligence and not those specific to educational attainment [46, 49].

The second of the three studies to appear found 148 loci (53 novel when it appeared online) associated with intelligence, with a sample size of 300,046 participants [31]. This study also used the UK Biobank VNR test and several other samples that formed a general intelligence component from three-or-more, mostly fluid intelligence-type tests. The third study to appear identified 205 loci (84 novel when it appeared online) using 269,867 participants [47]. This study was conditioned on socioeconomic status, and combined tests of cognitive ability and scores on scholastic aptitude tests.

It has become clear, therefore, that the genetic contribution to intelligence differences is highly polygenic, i.e. there are large numbers of independent genetic variants, each of which accounts for a tiny proportion of intelligence variation.

The three studies above used polygenic scores to provide out-of-sample predictions of intelligence based solely on DNA-SNP data [31, 46, 47]. A polygenic score is an individual-level predictor derived from the sum of effect alleles at a SNP, weighted by the regression co-efficient describing each SNP’s level of association with the trait, in this case intelligence. The polygenic scores predicted 4–7% of intelligence variance in independent samples; another study predicted 10.6% [50]. Thus, a blood sample at birth in these samples predicts intelligence with about the same effect size as parental socioeconomic status, i.e. they do not predict well; neither is of practical use for predicting the intelligence of an individual. The proportion of variance explained by polygenic scores rises with sample size, so the predictive power is likely to rise as sample sizes increase [51]. This raises ethical issues—outlined more elsewhere [52]—which should be addressed by well-informed professionals and lay people from appropriate interest groups and areas of expertise. We emphasise that the results above apply to the samples tested, all of which were of European ancestry, and relatively few of which, probably, were from very deprived situations. Therefore, results reported here may not be assumed to apply to other populations, or to the same populations at other times.

### After GWAS of intelligence: clues to mechanisms?

**Fig. 2:**
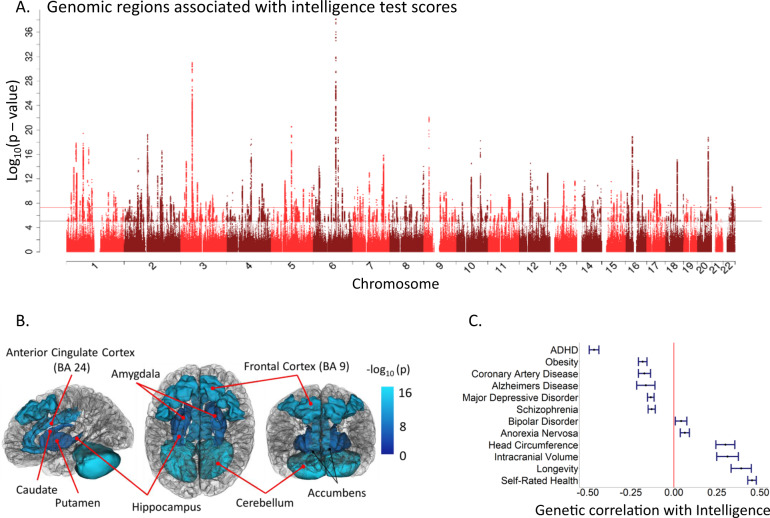
This shows genetic loci associated with intelligence test scores [46], intelligence’s overlap with transcription differences in the brain, and some of intelligence’s genetic associations with physical and mental health. **A** Manhattan plot displaying 187 regions of the genome associated with intelligence test scores. The chromosomes are on the *x* axis and the −log_10_ *P* value is on the *y* axis. Each dot represents a single nucleotide polymorphism. The horizontal red line indicates the genome-wide significant threshold of *P* = 5 × 10^−8^ and the horizontal black line represents genome-wide suggestive variants at *P* = 1 × 10^−5^. **B** The relationship between specific gene expression profiles in the cortex and intelligence-gene associations. Illustrated here is the finding that tissue-specific expression in and across the brain is associated with intelligence; the full list of associations is in the original report [46]. **C** Some of the genetic correlations between intelligence test scores and phenotypes linked to health, mental health, and measures of brain size [46]. A positive genetic correlation indicates that the genetic variants associated with higher intelligence test scores are associated with a greater value of the trait or a higher likelihood of developing the disorder. Longevity, intracranial volume, self-rated health, and head circumference all show positive genetic correlations with intelligence. A negative genetic correlation describes instances where the genetic variants associated with higher intelligence are also those that are associated with a lower value of the trait or a lower likelihood of developing the disorder. Traits such as ADHD, obesity, coronary artery disease, major depressive disorder, and Alzheimer’s disease show negative genetic correlations with intelligence.

Finding genetic loci whose variants are associated with intelligence differences only helps to understand these differences if we understand the mechanistic consequences of the genetic variation. GWAS data sets’ results on intelligence have found associations between SNP variation and tissue-specific gene expression across many of the brain’s cortical regions (Fig. 2) [31, 46, 47]. SNP variation associated with intelligence has been linked to tissue-specific gene expression in specific classes of neuron, including pyramidal neurons of the somatosensory cortex, the CA1 region of the hippocampus, midbrain embryonic GABAergic neurons, [53] and medium spiny neurons [47]. These associations indicate that, rather than any one specific area, the association between genetic and intelligence variation is probably mediated in part by individual differences in gene expression across the cortex.

Genetic variants associated with intelligence test scores tend to cluster in groups of genes linked with neurogenesis, the synapse, neuron differentiation, and oligodendrocyte differentiation [46]. These results are consistent with previous studies that found an association between intelligence and brain-expressed genes [54], as well as the genes expressed in the postsynaptic density and its associated components of the NMDA-receptor signalling complex [55] and Arc complex [56] more specifically. Together, these studies highlight the role of the synapse, and possibly the postsynaptic density and/or its associated components, as being biological systems that, when perturbed by common genetic variation, are associated with some of phenotypic differences in intelligence. However, the variance accounted for by the intelligence-associated SNPs found in these biologically plausible tissues is probably very small. Nevertheless, efforts to understand DNA versus intelligence phenotype associations at levels such as neuron types and gene systems are ways to tame the huge number of cognition-related SNPs, each of which has a miniscule effect size.

### Pleiotropic associations and intelligence

Pleiotropy (see Glossary) describes instances where variation at a region of the genome is associated with multiple phenotypes. Widespread pleiotropy between two phenotypes can be detected by deriving a genetic correlation (see Glossary) between the two phenotypes. A genetic correlation describes the average genetic effect shared between two traits as well as whether it is positive or negative; i.e. a positive genetic correlation occurs when genetic effects associated with an increase in one trait are also associated with an increase in a second trait, and a negative genetic correlation occurs when genetic effects associated with an increase in one trait are also associated with a decrease in a second trait. A genetic correlation co-efficient is derived using all SNPs from a GWAS regardless of the SNPs’ levels of association with a trait. Genetic correlations can be derived using two independent samples; this has the advantage that any genetic correlations found between intelligence and disease, for example, will not be due to individuals’ having the disease’s symptoms (i.e. if the intelligence GWAS has been conducted in healthy individuals). Polygenic scores can also make predictions across traits by deriving a polygenic score for intelligence and using it to predict health, or brain imaging traits, for example. However, these shared genetic associations—detected by genetic correlations or polygenic scores—can arise due to vertical pleiotropy, horizontal pleiotropy, or spurious pleiotropy, which we explain below [57].

Genetic correlations derived using GWAS data sets have demonstrated that genetic variants associated with higher intelligence test scores are, on average, also associated with, for example, longevity [46], better physical health [58], and more advantaged socioeconomic position [16, 50, 59]. Genetic variants associated with higher intelligence are more likely to be associated with lower levels of traits associated with mental health problems (Fig. 2) [60]. On the other hand, genetic variants associated with higher intelligence test scores are, typically, slightly positively associated with autism spectrum disorder and anorexia nervosa [46].

Mendelian randomisation (MR; see Glossary) studies move beyond associations between intelligence and health variables to seeking evidence that one phenotype might be causally related to another. MR results have indicated that intelligence and education probably have a bi-directional causal relationship [61], and that intelligence might have some causal association with, for example, Alzheimer’s disease that is independent of any protective effects of education [61]. Such results from MR should be interpreted cautiously as they can be biased by dynastic effects [62] known to influence education [36], which is highly genetically correlated with intelligence. The presence of dynastic effects violates the independence assumption of MR, as they induce a correlation between the environment in which a child is raised and their genetic inheritance.

Assortative mating, the tendency to select a partner based on heritable traits similar to one’s own, can bias the results of MR [63]. Education and intelligence are traits with evidence of assortative mating; there are reports of cross-spouse correlations of *r* = 0.40 for intelligence and *r* = 0.60 for education [64]. These contrast with measures of personality where correlations of *r* = 0.10 are found [65]. Biases from assortative mating can be induced by cross-trait assortative mating whereby, for example, more highly educated women might select partners who are taller, resulting in an apparent ‘finding’ that height is causally associated with education [63]. Bias due to dynastic effects and assortative mating can be controlled for by performing MR within families [66].

GWASs of educational attainment show high genetic correlations with intelligence (rg = 0.70–0.80 [46]) and have identified 1271 independent genome-wide significant SNPs [50]. Bioinformatic analyses of these data have identified associations with genes expressed in the brain and other cortical tissues as well as genes whose level of expression is elevated both pre- and postnatally. Furthermore, many of the genes identified encode proteins that are involved in synaptic functions such as synaptic plasticity, and neurotransmitter secretion, consistent with what has been identified for intelligence [46, 55, 67]. However, the use of education as a proxy phenotype for intelligence in genetic studies should be interpreted with caution. For example, whereas genetic correlations with schizophrenia indicate that the genetic variation that is associated with higher intelligence test scores is also associated with lower risk of schizophrenia, the genetic variants associated with attaining a longer and higher-level education are associated with higher risk of schizophrenia [46]. In a study investigating this phenomenon, SNPs associated with lower intelligence test scores, less education, and an increased risk for schizophrenia were also associated with early developmental processes [68]. This contrasted with the SNPs associated with lower education, and a lower risk of schizophrenia which were associated with biological processes of mature synapses.

A complementary explanation of some of the difference in genetic correlations between intelligence and education with schizophrenia focussed on “non-cognitive skills” [69]. The study used genomic structural equation modelling [70] to perform a GWAS by subtraction, resulting in associations specific to education once variance attributable to differences in intelligence was removed. The resulting GWAS of so-called “non-cognitive skills” found a heritability of 6.6% (SE = 0.2%), and a positive genetic correlation with schizophrenia of rg = 0.26 (SE = 0.02). However, whereas the cognitive traits captured by a GWAS of education were negatively genetically correlated with schizophrenia, the “non-cognitive traits” were genetically associated with a greater schizophrenia risk. A single cognitive performance test was used (UK Biobank’s VNR). This short test is unlikely to have captured all variance associated with intelligence and, so, this uncaptured cognitive ability variance would also be included in the “non-cognitive skills” (which would therefore be a misnomer) as evidenced by a genetic correlation of rg = 0.31 (SE = 0.06) with other measures of intelligence.

## Intelligence and the brain

### Intelligence and brain volume

There is a well-replicated, modest positive association between brain size and intelligence test scores. Brain size is usually measured as total volume, assessed in magnetic resonance imaging (MRI) scans. A meta-analysis of data from over 148 studies across more than 8000 individuals [71] estimated the association at *r* = 0.24. A re-analysis of those data including only healthy adults estimated the association at *r* = 0.31; this rose to *r* = 0.39 when it included only the studies judged to have used better-quality intelligence testing [72]. In a single sample of 18,426 middle- and older-aged participants of the UK Biobank (age range 44–81 years), the association between intelligence and total brain volume was estimated at *r* = 0.276 (95% CI = 0.252, 0.300) [73]. This is about halfway between the two previous estimates, and has the benefit of eliminating cross-cohort heterogeneity that can influence meta-analytic results.

There are many other ways to interrogate brain differences, beyond overall brain size, which, on its own, is not informative about the brain’s complexity. For example, the fact that there are substantial sex differences in brain size [74] but very small or no sex differences in mean intelligence [75, 76] is likely to be because multiple aspects of the brain’s structure, function, and connectivity are compensatory for any apparent brain size difference. Notably, there do not appear to be sex differences in the size of the brain-intelligence correlation [73, 74]. There is evidence that the magnitude of associations with intelligence vary as a function of brain tissue type and locus. A study that included brain cortical characteristics (volume, area, and thickness), total volume of subcortical structures, and measures of white matter macro- and micro-structure found that, together, they accounted for up to 18% of the variance in general intelligence in 73-year-olds [77]. In the wider age range of UK Biobank, multiple structural measures accounted for more of the variance in intelligence in older-age (13.6%) compared to middle-age participants (5.4%), only outperforming the single variable of total brain volume in the former group [73].

**Fig. 3:**
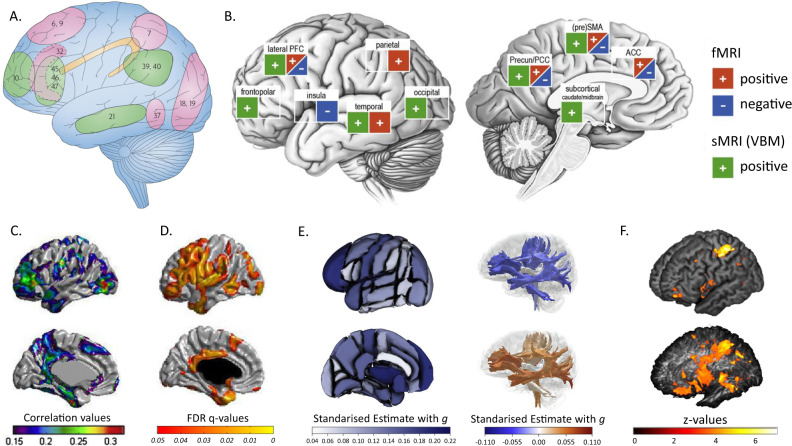
Possible brain loci of human intelligence differences. **A** The Parieto-Frontal Integration Theory (P-FIT) of intelligence differences, proposed by Jung and Haier [78]; reproduced with permission from [23]. **B** A meta-analysis of functional and structural (voxel-based morphometry only) studies of intelligence; reproduced with permission from [85]. **C** Associations between cortical thickness and intelligence in children (age range 6–18 years; *N* = 216); reproduced with permission from [83]. **D** Associations between cortical thickness and intelligence in older adults (age 73 years; *N* = 588); reproduced with permission from [79]. **E** Associations between intelligence test scores and regional cortical volume (left), and white matter tract fractional anisotropy (upper right, blue) and mean diffusivity (lower right, orange) in middle-aged and older adults (age range 44–81 years; *N*_range_ = 7201–18,426); reproduced with permission from [73]. **F** Associations between lesion locus and intelligence in cortical (top) and subcortical (bottom) loci (age *M* = 49, SD = 16 years; *N* = 241); reproduced with permission from [80].

The Parieto-Frontal Integration Theory attempted to summarise the intelligence-related brain regions implicated by structural, functional, and diffusion imaging studies of the brain [78] (Box 3). It identified that variations in the structure and function of lateral and medial frontal, parietal, lateral temporal, and lateral occipital cortex, and underlying white matter connectivity (such as the arcuate fasciculus) were associated with individual differences in intelligence. The available neuroimaging research at the time (37 studies) was hampered by small sample sizes, variable methods of measuring intelligence, and the-then limited number of diffusion MRI (dMRI) papers. This probably contributed to the relatively weak convergence of findings; even the most strongly implicated regions were supported by ≤60% of the papers surveyed. P-FIT theory receives support from some newer empirical findings (Fig. 3). We shall consider more recent studies in the light of it.

Box 3 Routes from the brain to theories of intelligence **P-FIT and beyond**

We mention the parieto-frontal integration theory (P-FIT) [78] in the main text as a trellis upon which to hang diverse research results relating brain measures to intelligence test scores. Here we describe other theories that are not necessarily aimed specifically at where intelligence differences reside in the brain (as was the P-FIT), but they are nonetheless informative of the neuroscience of intelligence differences. For example, a narrative synthesis of several divergent traditions within the functional brain imaging literature identified a number of regions consistently showing convergent activations across multiple executive function tasks [145]. There are variations in the nomenclature (e.g. Multiple Demand Network [MDN], fronto-parietal control network, the superordinate cognitive control network, and the extrinsic mode network [145]), yet these schemas commonly comprise dorso-medial and lateral prefrontal cortex, insula, and parietal cortex. The more recent extended MDN [145] also includes the putamen, thalamus, and more dorsolateral aspects of prefrontal cortex. Though these results are based on convergent functional activations across multiple tasks (rather than patterns of activations from a general factor of intelligence indicated by multiple tasks), the regions implicated from these functional studies bear a striking resemblance to the P-FIT. Integration of structural and functional data (along with other modalities) will be central to testing broader theories about the nature of intelligence. It will provide important biological constraints to questions about the degree to which intelligence is a single construct in the brain, or arises from a number of overlapping brain networks that support the variety of psychological processes that are required by the multiple tests from which intelligence is derived (e.g. [146]). It will also allow insights into the hypothesis that cortical connectivity facilitated by white matter is a biological substrate for individual differences in processing speed, which is hierarchically subordinate to fluid intelligence [92, 147].

**Contributions of longitudinal and lesion studies**

If intelligence is supported by a large distributed network of brain regions and their connections, there may well be many potential cerebral routes to intelligence differences, and to intellectual changes. This is a matter on which neuroimaging can offer important insights. Data from lesion studies and the ageing process can be seen as helpful in triangulating whether certain brain regions might be necessary for higher intelligence, rather than being related due to confounding factors (such as pre-existing, lifetime differences in brain volumes, in the example of ageing). Nonetheless, both methods are still limited.

Longitudinal studies offer a more stringent test of causal hypotheses than do cross-sectional studies. For example, if white matter health is important for higher intelligence, we would expect declines in both to be correlated [94]. However, even finding correlated changes between intelligence test scores and changes in a given brain region or pathway is still potentially confounded. A region that is not central to intellectual function could still exhibit correlated changes because its structural decline is simply correlated with regions that do support processes central to intelligence. Improved characterisation of longitudinal brain ageing will elucidate the degree to which this (the magnitude of correlated regional brain change) is an issue for localisation of intelligence.

Lesion studies offer valuable data on individuals in whom there has been a sudden and specific focal insult, which can be linked to differences in intelligence [80, 81]. Those brain loci more strongly linked to lower intelligence can allow mapping of the relative importance of specific regions. However, lesion studies may be limited by the numbers required to eliminate statistical power variability across the brain; i.e., the regional coverage of lesion loci in the sample might be heterogeneous. The fact that lesions often involve both grey and white matter is a further issue. Parsing their relative importance for a behavioural outcome (and identifying the underlying connective pathways affected in each case) at a particular locus requires many participants with selective (grey or white) tissue damage.

### Brain grey matter and intelligence

Associations between higher intelligence test scores and greater brain cortical volume and thickness in adults [73, 79], as well as data from lesion studies [80, 81] show stronger magnitudes across areas cited by the P-FIT (Box 3), though with consistently small effect sizes. The intelligence-cortical thickness relationship changes over the life course, with negative associations reported in 10-year-olds [82–84]. In the largest of these recent adult studies (*N* = 18,426, ages 44–81 [73]), some of the strongest associations were also found between intelligence and the volumes of the insula, posterior cingulate/precuneus (*r* < 0.20). These regions were not implicated in the initial P-FIT, but were identified in a meta-analytic update [78] using both functional and structural MRI data. However, considering only structural voxel-based morphometry analyses in easily-meta-analysable common space meant other types of structural study were excluded [85]. The large UK Biobank-based study [73] found that the intelligence associations with the volume of the thalamus (*r* = 0.25) were 1.5 times larger than for any other subcortical structure (*r* 0.06–0.17). The caudate, which had also previously been implicated in intelligence differences, albeit in smaller samples [86, 87], was also correlated with intelligence in the UK Biobank analysis (*r* ≈ 0.13), but only hippocampal (*r* = 0.05) and thalamic volumes (*r* = 0.19) showed unique associations with intelligence in a multivariate model including all subcortical structures. These findings are in line with the extensive cortical connectivity profiles of both insula and thalamus [88, 89].

### Brain white matter and intelligence

The last 20 years have witnessed an explosion of white matter brain imaging studies, due to the advent of dMRI. By exploiting the influence of various white matter properties (axonal myelination and diameter, among others [90]) on the motion of water molecular diffusion, dMRI enables inferences about white matter microstructure. Measures such as fractional anisotropy (FA; an index of the directional coherence of diffusion) and mean diffusivity (MD; the average magnitude of diffusion) are commonly used metrics. Both show general and regional ageing effects: on average, FA goes down with age, and MD goes up [91].

When assessed as summary measures across multiple parts of white matter, more directionally coherent water molecular diffusion (higher FA) and lower overall magnitude of diffusion (lower MD) are associated with higher intelligence test scores in studies together covering ages 8–81 years [73, 92, 93], typically with small effect sizes. FA and fluid cognitive ability show significantly coupled declines even over a 3-year period in older age (*r* = 0.31; [94]). Whereas we acknowledge inconsistencies in white matter tract nomenclature and identification, there is emerging evidence that long range cortico-cortical (association pathways), the genu (more so than splenium) of the corpus callosum, and subcortico-cortical (mainly thalamic) pathways show numerically larger associations with intelligence than projection fibres (though all *r* < 0.11; [73]). The identification of these pathways as associated with intelligence accords with the grey matter findings above, as these are broadly the connections that facilitate frontal, parietal, lateral temporal (and subcortical) interconnectivity. Age-related accrual of white matter damage (measured by white matter hyperintensities [WMHs]) is associated with lower (*r* = −0.106) and longitudinally coupled declines (*r* ≤ −0.334) in cognitive function [95, 96]. WMHs might interfere with selective pathways, because they tend to accumulate predominantly superior to the lateral ventricles [97].

Larger effect sizes for brain-intelligence associations can be found with multiple measures of white matter microstructure. One study derived a general factor of white matter integrity, across 12 white matter tracts, using three diffusion-based metrics: FA, longitudinal relaxation time, and magnetisation transfer ratio [92]. These three general factors showed only small correlations with each other, indicating that they might capture non-overlapping aspects of white matter microstructure. Together, they accounted for about 10% of intelligence differences in 73-year-olds. This link between white matter integrity and intelligence was fully mediated by the cognitive domain of processing speed.

It appears that the brain regions and their underlying connections that are associated with individual differences in our most complex cognitive abilities are also those which might: (i) show greater areal expansion as a function of increasing brain size [98]; (ii) be those that are latest to develop [99]; and (iii) be those most susceptible to brain ageing [91] and potential determinants thereof, such as vascular risk [100]. It is important to note that, even when brain regional measures are used, effect sizes remain relatively low, at an upper limit of ~*r* = 0.30 among well-powered studies; however, these estimates appear robust and replicable in large samples [73]. These findings illustrate the small but significant associations of multiple facets of brain structure with intelligence differences. This appears especially at older ages when a larger proportion of the variance in these measures is probably driven by differences in age-related neurodegenerative processes. Modest associations should not be surprising given the macro-scale of the brain variables, and that measures discussed above are only few of the potentially large number of brain properties that might be measured.

### Newer approaches to brain-intelligence associations

Emerging approaches have begun to model multivariate cross-tissue contributions to intelligence across selective grey matter regions and white matter pathways. For example, FA in the forceps minor and fronto-polar volume mediated 18.2% of the age association with fluid intelligence [101]. Such approaches have the potential to more directly test the specificity of brain-based network theories of intelligence, such as the P-FIT. Exploiting the brain’s structural connectome offers the chance to assess such network-based analyses with greater fidelity than via the measurement of fewer, larger pathways (though this brings a different set of limitations, e.g. [102]). Global measures such as connectomic efficiency [103], or variation in the ‘degree’ of nodes in morphometric similarity networks, have shown potential to predict intelligence differences (up to a remarkable 40% in one study with an *N* of 296 young adults [104]). Resting-state fMRI connectivity matrices predicted 20% of the variance in intelligence among young adults (*N* = 884; [105]). These results need replicating. It is therefore of interest to continue the development of clear and interpretable integration of structural and function brain connectomes [106] to inform our understanding of intelligence differences and the brain.

The next period of research should try to explain the associations between brain indices (which are sometimes rather crude) and intelligence, in addition to seeking and testing new brain variables. Having larger volumes of brain (and tissues therein) appears to be relatively strongly related to having greater numbers of neurons [107]. Cortical thickness differences are related to neuronal density, columnar arrangement, alongside dendritic arbour and glial properties [108]. Another promising metric may be the diffusion characteristics of grey matter, assessed using neurite orientation dispersion and density imaging, which are putative markers of dendritic density and arborisation and exhibit some P-FIT-like regional associations with intelligence (|*r* | <0.25) [109]. Yet, much work is required before we understand whether and how such specific microscopic features (some of which are estimated with neuroimaging) might specifically give rise to intelligence differences. Whereas new methods, larger sample sizes, and out-of-sample prediction designs have the potential to add further insights and complementary contributions to intelligence variation, we judge it will be important to: (i) test the incremental validity of newer measures beyond more conventional metrics (e.g. [110]); (ii) be critical regarding whether the newer measures offer more biologically tractable variables for understanding brain differences; and, if they do, (iii) integrate these new indices with other levels of explanation, bridging the spectrum of macrostructure to cellular.

## Toward a better integration of genes, brains, and intelligence

We structured this review into sections on phenotypic issues about intelligence, the genetic associations with intelligence test scores, and their brain correlates. This partly reflects the division of labour among the authors, but also reflects the current prevailing separation of these lines of enquiry. We should have liked there to have been more amalgamated brain-cognition-genetics studies, and we should have liked more integrated sections, examples and ideas to review. However, we consider that to be a lesson for us, and for the field, about the sorts of studies that could be done. These divisions are also in part because research on the statistical phenotypic nature of intelligence has been going on for more than a century. Genetics and brain imaging are relative youngsters, and methodologically fast-moving. New methods have blossomed in these fields, and debates and enquiry into the merits of these will continue apace. To mention a few examples, there are debates about both the diffusion and fMRI work in terms of cross-lab stability for neuroimaging [111, 112], and there are discussions about the causal nature, if any, of the observed genetic correlations between intelligence and other phenotypes, as well as the role of non-transmitted genetic effects. The phenotypic nature of intelligence, too, has areas of contention: some areas of the neuroscience and cognitive literature focus on experimental cognitive tasks that assess aspects of so-called executive functioning, in contrast to the psychometric tests upon which intelligence scores are based. Consequently, reviews that focus solely on, say, executive function(s) or working memory might not fully bring out their strong phenotypic [8, 113–115], genotypic [116, 117] and neurostructural (Box 3) overlaps with psychometric intelligence. It would be of interest to develop a theoretical superstructure to unite these cognitive disciplines; it is unfortunate that cognitive neuropsychologists and psychometricians do not bring their tests and constructs together more when, empirically, they are strongly related [114]. In this section we summarise some recent studies that have integrated genes and brains to try to understand intelligence differences, before discussing their implications and potential future directions for a more integrative and nuanced account of some biological underpinnings of intelligence differences.

### Looking at all three of genes, brains, and intelligence

Genetic correlations find that the genetic variants associated with intelligence are shared in part with those associated with volumetric measures of brain structure, such as intracranial volume (rg = 0.27), total brain volume (rg = 0.23), grey matter volume (rg = 0.08), white matter volume (rg = 0.08) [31], and volume of the left posterior cingulate cortex (rg = 0.23) [118]. Positive genetic correlations have been identified between healthier brain white matter microstructure and higher intelligence [119]. Reaction time—in which higher values are worse—had genetic correlations of −0.18 on average with brain white matter health/integrity, based on FA, as well as widespread positive genetic correlations of 0.17, on average, with axial diffusivity, MD, mode of anisotropy, and radial diffusivity [119].

A study which found that a polygenic score for intelligence predicted 3–5% of intelligence differences in a new sample also reported that this association was partially mediated to a small extent by brain cortical thickness and surface area of the anterior cingulate cortex, the prefrontal cortex, the insula, and the medial temporal cortex [120]. The results are consistent with the notion that the genetic variants’ associations with intelligence test scores might be accounted for partly through their associations with variation in the structure of the brain in many of the areas already linked in phenotypically with intelligence differences.

The high level of polygenicity of both intelligence [46] and structural brain measures [118, 119] may indicate that a wealth of biological systems are associated with individual differences in both. This may prove an obstacle for uncovering a mechanistic account of how genetic variation is associated with brain and intelligence differences, as any biological system associated with both is likely to explain only a fraction of phenotypic variance in both brain and intelligence measures. However, despite the small effect sizes likely to be associated with each mechanism separately, future work aiming to produce a more mechanistic account of intelligence differences should examine the relative importance of any biological system identified, as well as examining its association with brain imaging measures.

The pleiotropy identified between intelligence and cortical measures also requires further examination. Specifically, the relative importance of vertical pleiotropy and horizontal pleiotropy in the generation of genetic correlations between cognitive ability and brain variables is currently unknown. By understanding the forms of pleiotropy responsible for such genetic correlations, more mechanistic accounts of intelligence differences can be formulated. For example, should vertical pleiotropy drive the genetic correlation between intelligence and brain structure then it could indicate that brain structure is causal in intelligence differences; however the opposite scenario is possible, i.e. where intelligence partly drives brain differences. Should these genetic correlations be the result of horizontal pleiotropy this would indicate that the same genetic loci are associated with both brain structure and intelligence with no causal relationship between the two.

A related difficulty in the interpretation of genetic correlations between intelligence and brain morphology arises from non-transmitted genetic effects [36, 37]. Here, genetic correlations between intelligence and cortical measures may, in part, be the result of the genotype of the parent being linked to rearing practices that support both healthy brain development, and intellectual growth. Within-family GWAS will provide future opportunities to identify these effects [66] and gauge the magnitude of the residual genetic relationship between intelligence and brain structure, in the absence of the effect that parental genotype may have on both these variables.

The finding of a genetic *g* factor [29] also has implications for the functional annotation of the loci identified as being associated with intelligence. Specifically, loci found to be associated with cognitive abilities might be associated with the variance from a general factor or they might be associations specific to the domain examined, or even to the specific test within the given domain. This can be seen most clearly when examining genetic loci previously associated with the Trails B cognitive test; it was shown [29] that Trails B’s scores’ associations with genetic loci were shared with other tests of cognitive ability, and so were more general than initially assumed. This is a potential issue for examining more mechanistic accounts of intelligence differences, as variance that is both common across cognitive tests, and the variance that is specific to any particular testing domain or single test might be included together. By using techniques such as genomic SEM to distil these associations into those that are general across cognitive domains, and those that are specific to each domain or test could help in identifying plausible biological mechanisms linked with each.

### Beyond just *gen*-‘omics’

There are, as yet, few studies that directly integrate genetic variation, brain imaging indices, and intelligence test scores. We barely have even a rudimentary understanding of how variation in the huge number of genetic variants identified as being associated with intelligence test scores and brain indices might directly relate to mechanisms (e.g. protein expression) and how these go on to facilitate the neurobiological machinery whose properties can be estimated and tested for any associations with differences in general cognitive ability. The explanatory gap between genetic loci and cognitive test scores is massive, and liable to induce a range of responses from the pleasure and terror of the Burkean sublime (a job to be done, though daunting), to less helpful routes of either premature and simplistic reductionism, or hopelessness.

There are fields that might help to bridge the gap, and contribute toward understanding intelligence differences, such as epigenetics [121, 122], transcriptomics, proteomics, virtual histology, and others. Compared with, say GWAS methods, these are newer still, and will take time to even partly fill in the spaces between the triumvirate of genes, brains, and intelligence. However, genome-wide methylation studies examining intelligence and brain structure have begun to show some converging results. An association was identified between cg12507869 on chromosome 10 in the INPP5A gene and cognitive ability, as measured using the Mini-Mental State Examination, and with phonemic verbal fluency [121]. Methylation probes in the INPP5A gene have also been shown to be associated with hippocampal volume, cg25594319, although this did not withstand correction for multiple testing across the brain regions examined [122]. Nevertheless, we judge that poly-epigenetic approaches (e.g. EWAS and resultant epigenetic scores created out-of-sample) and larger sample sizes, particularly in epigenetic-neuroimaging studies [123] will yield further progress. For example, epigenetic signatures of smoking, mortality, and inflammation exhibit cognitive associations and regional cortical correlates in older age that overlap with P-FIT areas [124–126].

‘Virtual histological’ methods—which exploit histological data on regional gene expression [127]—also have potential for understanding how genes and brain measures relate to each other in the context of cognitive differences. Unifying information on (i) gene expression patterns and (ii) brain structural information at the same specific spatial level (e.g. cortical parcels) allows researchers to ask whether brain regional differences in brain structure-expression associations are relevant for other correlates of brain structure, using a correlation-of-correlations approach. For example, those brain cortical regions that showed a stronger relationship between greater triacylglycerol expression and greater cortical thickness were also the regions where thickness was more strongly associated with general cognitive ability [128]. Multivariate and systems-specific approaches (which consider multiple proteins together) will further illuminate the unique contributions of specific proteins and the underlying mechanisms via which they might relate to the underlying neurobiological bases of cognitive differences.

Finally, we want to be clear about ‘prediction’ of intelligence using genetic or brain imaging variables. We advocate testing replication across samples; that is, if a set of genetic variants or one or more brain imaging variables is associated with psychometric intelligence in a discovery sample then it is prudent to test whether the association holds in replication samples. This is a means to validating and understanding the generalisability of the genetic and cerebral correlates of intelligence from a starting sample. Thus, replication via out of sample prediction into another sample is a tool via which we can enhance our understanding of reasons why people differ in their cognitive abilities. On the other hand, prediction of the intelligence of an individual from genetic or neuroimaging variables is not a practical or, in our view, desirable aim.

## Conclusion

Brain imaging and genetic associations with intelligence test score differences made progress in the last 10 years, with a raft of results based on new methods and large samples. Imaging and genetic variables account for a minority of intelligence variation. In both fields we conclude that: additional sources of variation should be sought; there is still a large explanatory gap separating us from even a partial mechanistic account of why people differ in intelligence; and the associations should not be taken to mean that there are immutable contributions to intelligence. When, or maybe if, we understand these and future associations, there might be hints as to what tends to make optimal cognitive development and healthy cognitive ageing. We recognise and encourage research on other substantial sources of variation in intelligence, social as well as biological [129].

## Glossary

Candidate gene design: This design focusses on variants in specific genes that are thought to be linked to the trait of interest due to the biological functions of the genes and genetic variants that are selected.
Genetic correlation: This describes the standardised average genetic effect shared between two phenotypes. It is a correlation between the tested genetically heritable elements of each trait.
Genome-wide association study (GWAS): This study design is used to identify loci throughout the genome associated with a trait or disease state. GWAS can include millions of mostly common single nucleotide polymorphisms from across the genome. These are each tested for association with the phenotype of interest. The problem of type 1 statistical errors in such a large number of tests is controlled for by adopting a stringent *p* value cut off, typically of 5 × 10^−8^.
Heritability: This is the proportion of phenotypic variance that is attributable to the variance found in genetic factors. In the context of GWAS it is typically only the additive genetic factors that are considered from variants that are in linkage disequilibrium (see below) with the common SNPs used, and so the heritability estimate is the narrow-sense heritability.
Linkage disequilibrium (LD): This is the non-random association of alleles located in at least two distinct genetic loci.
Mendelian randomisation (MR): MR is a statistical technique that uses genetic variants, which are unchanged from conception, to test for possible causal relationships between an exposure and an outcome. MR typically uses SNPs that have attained genome wide significance for an exposure trait, such as smoking, as proxy variables for the exposure trait, i.e. forming an instrument for it. Such instrumental variables are defined by three key assumptions. First, that they are associated with the exposure. Second, they are associated with the outcome only through the exposure. Third, that they are not associated with confounders with respect to the outcome.
Pleiotropy: This describes the finding that multiple phenotypes can be associated with genetic variation at the same locus. First, this may be due to the locus’s having an independent causal effect on each phenotype, which is termed horizontal or biological pleiotropy. Second, it can describe instances whereby one phenotype is causally associated with a second phenotype, meaning that any genetic associations found with the first phenotype will be shared with the second; this is termed vertical or mediated pleiotropy.
Single nucleotide polymorphism (SNP): SNP is pronounced ‘snip’. This is the most common type of genetic variation between people. Each SNP is a substitution of a single nucleotide at a specific position in the genome. In a GWAS, a SNP is treated as the statistical unit of association.
